# Factors influencing the time to surgery after neoadjuvant chemotherapy in breast cancer patients

**DOI:** 10.1007/s00404-020-05494-6

**Published:** 2020-03-14

**Authors:** Carolin Müller, Ingolf Juhasz-Böss, Gilda Schmidt, Peter Jungmann, Erich-Franz Solomayer, Georg-Peter Breitbach, Stephanie Juhasz-Böss

**Affiliations:** Department of Gynecology, Obstetrics and Reproductive Medicine, Saarland University Medical Center, Kirrbergerstraße 100, 66424 Homburg/Saar, Germany

**Keywords:** Breast cancer, Neoadjuvant therapy, Time to surgery, Delaying factors

## Abstract

**Purpose:**

It is suspected that delayed surgery after neoadjuvant chemotherapy (NACT) leads to a worse outcome in breast cancer patients. We therefore evaluated possible influencing factors of the time interval between the end of NACT and surgery.

**Methods:**

All patients receiving NACT due to newly diagnosed breast cancer from 2015 to 2017 at the Department of Gynecology, Saarland University Medical Center, were included. The time interval between end of NACT and surgery was defined as primary endpoint. Possible delaying factors were investigated: age, study participation, outpatient and inpatient presentations, implants/expander, MRI preoperatively, discontinuation of chemotherapy, and genetic mutations.

**Results:**

Data of 139 patients was analyzed. Median age was 53 years (22–78). The time interval between end of NACT and surgery was 28 days (9–57). Additional clinical presentations on outpatient basis added 2 days (*p* = 0.002) and on inpatient basis added 7 days to time to surgery (*p* < 0.001). Discontinuation of NACT due to chemotherapy side effects prolonged time to surgery by 8 days (*p* < 0.001), whereas discontinuation due to disease progress did not delay surgery (*p* = 0.6). In contrast, a proven genetic mutation shortened time to surgery by 7 days (*p* < 0.001). Patient’s age, participation in clinical studies, oncoplastic surgery, and preoperative MRI scans did not delay surgery.

**Conclusion:**

Breast care centers should emphasize a reduction of clinical presentations and a good control of chemotherapy side effects for breast cancer patients to avoid delays of surgery after NACT.

## Introduction

Breast cancer is the most frequent malign disease in women. Lifetime risk adds up to 1:8 [1] and the incidence of breast cancer has even increased over the last years so that in 2020 about 77,600 new diseases may be expected in Germany. Breast cancer mortality has dropped since 1990 thanks to many new successful therapy strategies [1]. One of the therapy strategies is the neoadjuvant chemotherapy (NACT) which allows disease downstaging and higher rates of breast-conserving therapy [2]. According to German guidelines, the operation following NACT should be performed 2–4 weeks after the end of NACT to bypass the leukocyte nadir [3]. There is compelling evidence that a delayed therapy onset of adjuvant chemotherapy worsen outcome [4, 5], which was also shown for neoadjuvant settings [6]. Sanford et al. reported worse outcome when time to surgery exceeds 8 weeks after NACT [6]. Several factors might influence this time interval, and especially prolonging factors are of considerable interest to improve clinical pathways.

We therefore determined the time interval between the end of NACT and surgery, and investigated possible influencing factors: age, study participation, outpatient and inpatient presentations, implants/expander, MRI preoperatively, discontinuation of chemotherapy, and genetic mutations. We previously reported influences on the onset of NACT based on the same register [7]. Onset of NACT was delayed by additional clinical presentations, making it particularly interesting, whether this also applies to the time between NACT and surgery.

## Patients and methods

### Data collection

Data were collected of all patients receiving a neoadjuvant chemotherapy (NACT) at the Department of Gynecology, Saarland University Medical School from 2015 to 2017. Therapy schedule was based on diagnosis and determination of prognostic factors. The primary endpoint was the time interval between the end of NACT (date of last infusion) and date of surgery.

Patient’s age, stage, and tumor biology were recorded. The type of operation was documented (breast-preserving therapy versus mastectomy and reconstruction with breast implants or expander). Chemotherapy plans were based on clinical studies or standard therapy was applied. From 2015 to 2017, the following neoadjuvant study designs were addressed: GAIN II, GeparOcto, GeparX, Nadens, and Adapt studies. The number of presentations in the hospital was recorded, either on outpatient or inpatient basis. It was noted whether there was a genetic mutation or whether additional imaging (for example MRI) had to be performed before surgery. Furthermore, discontinuation of chemotherapy and reasons for discontinuation (therapy complications/disease progress) were recorded.

### Data management and statistics

Patient’s data were reviewed in the hospital’s digital documentation system (SAP, Walldorf, Germany). Data was collected using Microsoft Excel 2010^®^ (Microsoft, Redmond, USA). Further statistics were performed with SPSS 24.0 (IBM, Armonk, USA). Quantitative parameters like time intervals, patient’s age, and number of clinical presentations are presented as median and range. Qualitative parameters like tumor biology, type of operation (breast-conserving therapy, mastectomy or reconstruction with breast implants or expander), and other influencing factors are given as frequencies. Univariate regression analysis was performed, with bootstrapping of 100,000 samples, to determine the influence of the following factors on the time interval between the end of NACT and surgery: age, study participation, clinical presentations, reconstructive surgery (implants/expander), preoperative MRI, discontinuation of chemotherapy (therapy complications/disease progress), and genetic mutation. All procedures performed in the study involving human participants were in accordance with the ethical standards of the institutional and/or national research committee and with the 1964 Helsinki declaration and its later amendments or comparable ethical standards. Informed consent was obtained from every individual participant included in the study.

## Results

A total of 152 patients were scheduled for NACT between 2015 and 2017. After exclusion of 13 patients with insufficient records, 139 patients proved to be evaluable. Overall median time between the end of NACT and surgery was 28 days (9–57). The patient’s median age was 54 years (22–78).

Table 1 illustrates tumor and therapy characteristics. About half of the patients had a complete remission (ypT0) after NACT. None of the patients had primary metastasis. The most common surgical procedure was breast-conserving therapy (65%). The luminal A subtype was rare (4%), whereas luminal B, Her2-positive and triple-negative subtypes were equally distributed (32, 34, 30%). Genetic mutation was rare (4%). 90 patients (65%) were treated with standard chemotherapies according to guidelines and 49 (35%) received study therapies. The major part (47%) was treated according to the GAIN II protocol (23 patients) [8] followed by GeparOcto (18 patients) [9]. A total of 27 patients (19%) discontinued chemotherapy. Most of the patients discontinued chemotherapy due to side effects (20 patients) and some due to disease progress (4 patients). Therapy complications were polyneuropathy in nine patients, changes of laboratory values (for example increased liver value) in four patients, skin lesions (for example erythema) in three patients, port infection in one patient, and discontinuation of chemotherapy due to general deterioration in three patients.

**Table 1:** Tumor and therapy characteristics

	Absolute frequencies (*n*)	Cumulative frequencies (%)
*Staging*		
TNM-stage		
ypT		
0	74	53.2
1	40	28.8
2	11	7.9
3	6	4.3
4	4	2.9
X	4	2.9
ypN		
0	45	32.4
1	20	14.4
2	11	7.9
3	2	1.4
X	61	43.9
pN (sentinel node)		
0	65	46.8
1	7	5.0
X	67	48.2
M		
0	139	100
*Surgery*		
Type of surgery		
Breast-conserving therapy	90	64.7
Subcutaneous mastectomy	14	10.1
Simple mastectomy	35	25.2
Implants/expander	19	13.6
*Histology*		
Histological subtype		
Luminal A ER ± , PR ± , Her2neu neg, Ki67 ≤ 15%	6	4.3
Luminal B ER ± , PR ± , Her2neu neg, Ki67 > 15%	45	32.4
Her2 like ER ± , PR ± , Her2neu +	47	33.8
Triple negative	41	29.5
*Discontinuation of chemotherapy*		
Reasons		
Side effects of chemotherapy	20	14.3
Disease progress	4	2.9

Between the end of chemotherapy and operation, the patients had a median of 1 (0–7) outpatient presentations and a median of 0 (0–2) inpatient presentations. The major reasons for clinical presentations were planning of surgery (98%) followed by side effects of chemotherapy (17%), which is consistent with main reasons for repetitive appointments due to planning of surgery (29%) and due to side effects of chemotherapy (11%). In rare cases, a preoperative MRI (14%) or echocardiography (9%) was performed before operation (Table 2).

**Table 2:** Reasons for clinical presentations between neoadjuvant chemotherapy and surgery

	Absolute frequencies (*n*)	Cumulative frequencies (%)
*Planning of surgery*		
Number of presentations		
1	114	81.4
2	20	14.3
3	1	0.7
4	1	0.7
5	1	0.7
*Side effects of chemotherapy*		
Number of presentations		
1	15	10.7
2	6	4.3
3	1	0.7
5	1	0.7
7	1	0.7
All	24	17.1
*Further diagnostics*		
Echocardiography	12	8.6
Imaging (MRI, CT)	19	13.6
*Main reason for repetitive appointments*		
All	56	40
Planning of surgery	40	28.6
Side effects of chemotherapy	16	11.4

Clinical presentations on outpatient and inpatient basis considerably prolonged time to surgery. Every outpatient presentation added 2 days and every inpatient presentation added 7 days to the time to surgery. Discontinuation of NACT due to side effects also prolonged time to surgery by an average of 8 days, whereas discontinuation due to disease progress had no significant influence. A proven genetic mutation shortened the time to surgery by an average of 7 days. Age, study participation, oncoplastic surgery (implants / expander), and preoperative MRI did not delay surgery (Table 3).

**Table 3:** Influences on time to surgery assessed by univariate regression analysis

Parameter	Regression coefficient	95% confidence interval	*p*
Age	0.007	−0.1 to 0.1	0.054
Study participation	0.5	−2.4 to 3.5	0.72
Outpatient presentations	2.2	0.8 to 3.6	0.002
Inpatient presentations	6.9	3.9 to 10.3	< 0.001
Implants/expander	3	−1.4 to 7.6	0.189
MRI (preoperative)	3.8	−0.8 to 8.4	0.105
Discontinuation of chemotherapy (side effects)	8.2	3.7 to 12.6	< 0.001
Discontinuation of chemotherapy (disease progress)	−1	−5.7 to 2.7	0.622
Genetic mutation	−6.6	−8.8 to −4.5	< 0.001

## Discussion

Median time between NACT and surgery was 28 days in accordance with current German guidelines which recommend surgery 2–4 weeks after NACT [3]. Similar results were reported in a retrospective analyses of 1101 patients with 33 days between NACT and surgery [6]. Additionally, outcome seems to worsen when surgery is performed more than 8 weeks after NACT [6].

Worse outcomes by a delayed therapy onset was also shown for adjuvant settings. Especially patients with stage III of breast cancer, trastuzumab-treated Her2-positive tumors and triple negative carcinomas had inferior outcomes when time to chemotherapy was longer than 60 days compared to a therapy onset below 30 days after surgery [4]. Consistently, recurrence-free survival and overall survival decreases in patients with triple-negative breast cancer when chemotherapy started more than 30 days after surgery [5]. However, these studies investigated adjuvant chemotherapy settings, where chemotherapy is performed after surgery. There is still missing data for limits of the time between NACT and surgery. However, therapy centers should stick to the recommended range of 2–4 weeks [3], as worse outcome can be expected if surgery is delayed [6].

To provide possible approaches to improve patient care, we analyzed possible influences on time to surgery after NACT. Additional clinical presentations prolonged time to surgery—outpatient presentations by 2 days and inpatient presentations by 7 days. The most common reason for a clinical presentation was planning of surgery. Most of the patients were seen after completion of chemotherapy to plan surgery, but some (2%) were seen earlier when regular presentations for sonographic controls were already scheduled close to the end of chemotherapy—this is not standard of care but saves time and resources. This might be a considerable approach to minimize clinical presentations. Especially patients with complicated cases could already be advised during clinical follow-ups of chemotherapy. The second most common reason for a clinical presentation were side effects of chemotherapy. A good control of chemotherapy side effect is thus recommendable. For example, by routine antiemetic prophylaxis or GCSF (granulocyte colony stimulating factor) preparations. Taken together, breast care centers should optimize clinical pathways and the prevention of chemotherapy side effects to minimize clinical presentations and accelerate time to surgery.

Patient’s age had no influence on time to therapy onset, nor did study participation (Table 3). A possible reason might be that patients participating in clinical studies had the most part of administrative and structural restraints (like checking of inclusion/exclusion criteria, control of morphologic and biologic assessment, etc.) already done before NACT. One might expect that this delays the onset of NACT, but our previous analysis based on the same register showed no delay of NACT by study participation [7].

Oncoplastic surgery did not prolong time to surgery. Oncoplastic surgery offers a further approach for extending breast conserving surgery possibilities, with wide excisions and aesthetic results [10]. Although oncoplastic surgery is performed more frequently in patients with larger or multifocal tumors, there is no increased rate of local recurrences or re-operations [11]. Taken together, there is no reason to forgo oncoplastic surgery in terms of outcome and time to surgery.

Preoperative imaging (MRI scan) seemed to delay time to surgery on average by 4 days without reaching statistical significance (Table 3). This might be due to the limited number of patients. Previous studies reported delays of 11 [12] or even 22 days [13]. However, both studies were done in adjuvant therapy settings. The need for a preoperative MRI might be already clear during NACT, presumably allowing timely arrangements. Nevertheless, available data suggests that structural and organizational conditions should be optimized to minimize a possible delay of surgery by preoperative MRI.

The discontinuation of chemotherapy due to chemotherapy side effects prolonged time to surgery by 8 days. The majority of the patients stopped chemotherapy because of intolerable side effects, whereas a minority had disease progress. Patients suffering from side effects may have had to recover prior to surgery, presumably delaying time to surgery. In contrast, discontinuation of chemotherapy due to disease progress did not delay time to surgery. This seems to be preferable, as patients with disease progress need timely surgical treatment.

Time to surgery was shortened by 7 days, if genetic mutation was confirmed. Since patients with proven BRCA mutation are more likely to develop breast cancer, primary mastectomy is usually advised [14]. Neoadjuvant settings give enough time for genetic counseling and testing, so that the result will already be available before completion of chemotherapy. Thus, the recommended surgical procedure is already clear before NACT is completed, making it apparent that time to surgery is shortened.

Our study has some limitations. This study was performed in a single certified tertiary breast care center. Clinical pathways might differ in other centers. Sentinel-node-biopsy before NACT was standard of care in Germany during the study period. 72 patients (52%) in this study received SNB prior to NACT. With positive pretherapeutic lymph nodes (sonographic or according to SNB), an axillary lymphadenectomy was added to the surgical treatment after completion of chemotherapy. Nowadays, sentinel-node-biopsy is performed after NACT, which may additionally prolong time to surgery. Furthermore, sample size is small, and thus regression analysis had to be performed with bootstrapping. At least, not all possible delaying factors were addressed in this study. For example, some patients might ask for a second opinion before surgery.

## Conclusion

Median time between NACT and surgery is 28 days. To avoid delays of surgery, breast care centers should emphasize a reduction of appointments for clinical presentations of patients with breast cancer after NACT. A good control of chemotherapy side effects is recommendable, as this is the major reason for chemotherapy discontinuation which delays surgery. Earlier clarity about the surgical procedure seems to shorten the time to surgery for patients with proven genetic mutation, thus early planning of surgery should be emphasized. At least, patient’s age, participation in clinical studies, oncoplastic surgery, discontinuation of chemotherapy due to disease progress, and preoperative MRI scans seems not to delay surgery.
